# Cutting edge of endoscopic ultrasound-guided fine-needle aspiration for solid pancreatic lesions

**DOI:** 10.1007/s10396-023-01375-y

**Published:** 2023-11-01

**Authors:** Takuya Ishikawa, Kentaro Yamao, Yasuyuki Mizutani, Tadashi Iida, Hiroki Kawashima

**Affiliations:** 1 Department of Gastroenterology and Hepatology, Nagoya University Graduate School of Medicine, 65 Tsurumai-Cho, Showa-Ku, Nagoya, 466-8560 Japan; 2 Department of Endoscopy, Nagoya University Hospital, Nagoya, Japan

**Keywords:** EUS-FNA, Solid pancreatic lesions, Macroscopic on-site evaluation, Artificial intelligence, Comprehensive genomic profiling

## Abstract

This article provides an extensive review of the advancements and future perspectives related to endoscopic ultrasound-guided tissue acquisition (EUS-TA) for the diagnosis of solid pancreatic lesions (SPLs). EUS-TA, including fine-needle aspiration (EUS-FNA) and fine-needle biopsy (EUS-FNB), has revolutionized the collection of specimens from intra-abdominal organs, including the pancreas. Improvements in the design of needles, collection methods, and specimen processing techniques have improved the diagnostic performance. This review highlights the latest findings regarding needle evolution, actuation number, sampling methods, specimen evaluation techniques, application of artificial intelligence (AI) for diagnostic purposes, and use of comprehensive genomic profiling (CGP). It acknowledges the rising use of Franseen and fork-tip needles for EUS-FNB and emphasizes that the optimal number of actuations requires further study. Methods such as the door-knocking and fanning techniques have shown promise for increasing diagnostic performance. Macroscopic on-site evaluation (MOSE) is presented as a practical rapid specimen evaluation method, and the integration of AI is identified as a potentially impactful development. The study also underscores the importance of optimal sampling for CGP, which can enhance the precision of cancer treatment. Ongoing research and technological innovations will further improve the accuracy and efficacy of EUS-TA.

## Introduction

Endoscopic ultrasound-guided fine-needle aspiration (EUS-FNA) is a procedure in which a fine needle is inserted into a target lesion using endoscopic ultrasound (EUS) to collect specimens. In 1992, Vilmann et al. [1] reported the world’s first case of pancreatic cystadenoma diagnosed using EUS-guided biopsy. In Japan, the procedure was covered by insurance as EUS-FNA in 2010 and is now widely used to collect specimens from the pancreas and other intra-abdominal organs. In the field of pancreatic diseases, in particular, the importance of pathological diagnosis via EUS-FNA is increasing with the advent of new treatments and tests such as preoperative chemotherapy, immune checkpoint inhibitors, and comprehensive genomic profiling (CGP).

**Fig. 1:**
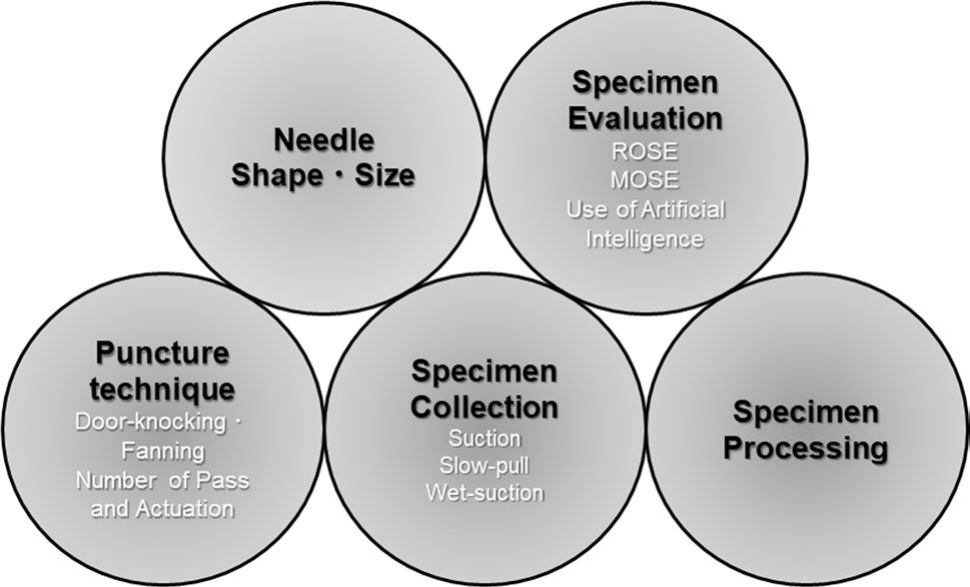
Various factors that can affect the diagnostic performance of EUS-FNA. *ROSE* rapid on-site evaluation, *MOSE* macroscopic on-site evaluation

There are various factors that can affect the diagnostic performance of EUS-FNA, including needle shape, puncture technique, specimen collection method, evaluation method, and specimen processing (Fig. 1). In recent years, several “core needles” for histological diagnosis have been developed in contrast to the conventional FNA needle for cytological diagnosis, and EUS-FNA mainly used for tissue collection is now called EUS-guided fine-needle biopsy (EUS-FNB), which is attracting attention for its usefulness. Currently, EUS-FNA and EUS-FNB are sometimes referred to together as strategies of EUS-guided tissue acquisition (EUS-TA) [2].

In this article, we review the efforts to improve the diagnostic performance of EUS-TA for solid pancreatic lesions (SPLs) based on the literature and discuss future prospects for higher-quality specimen collection. This article, however, is neither a systematic review nor a meta-analysis as the references were not systematically researched sufficiently to publish as a meta-analysis.

## Evolution of needles

**Fig. 2:**
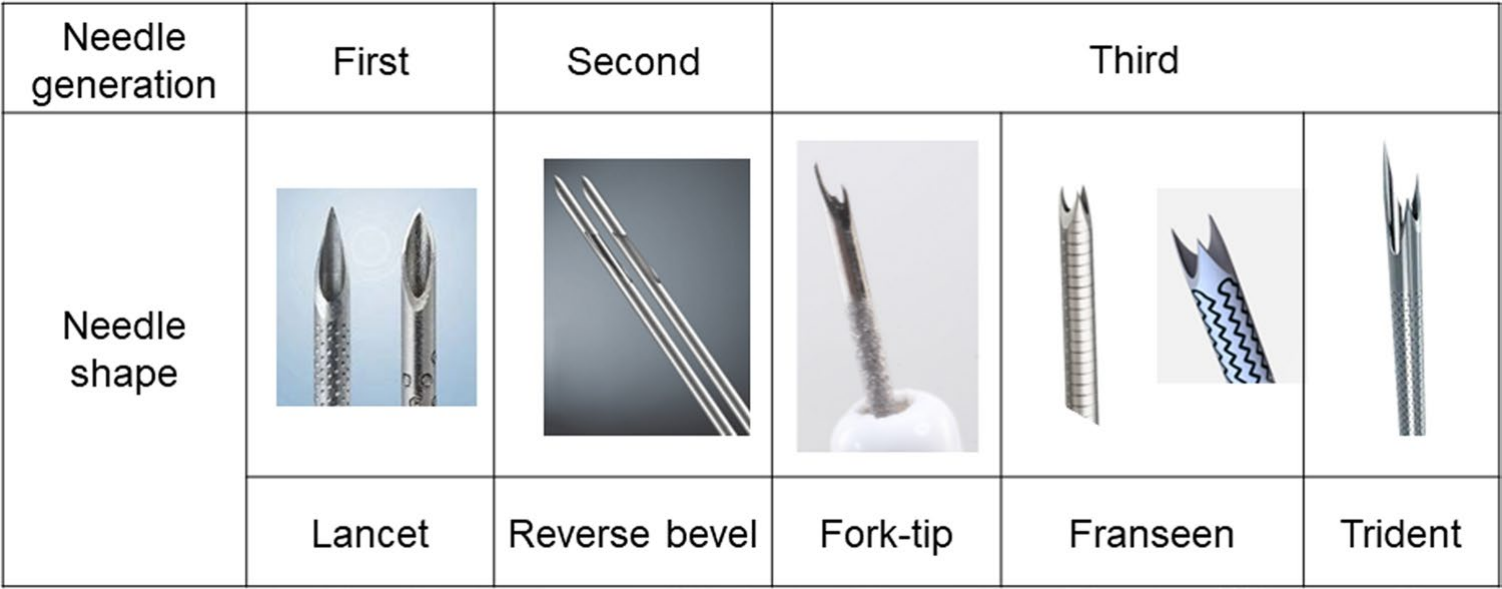
Changes in needle shape in each generation

In recent years, needles have been reshaped and evolved to collect a larger volume of specimen. Starting with the first generation of lancet-shaped needles, the second and third generation of core needles have been developed for EUS-FNB (Fig. 2). There have been many reports on the comparison of standard FNA and core needles [3–8]. Although there were some differences in the protocols of these studies, basically all of them reported that the diagnostic accuracy of EUS sampling for SPLs using standard FNA and core needles seemed comparable but that the core needle was superior in terms of overall histological specimen quality. Subsequently, several randomized studies comparing the core needles in patients with SPLs have been performed to date (Table 1). Karasenti et al. performed a multicenter, randomized, crossover trial comparing EUS-FNB with a 20G reverse-bevel needle (ProCore; Cook Medical, Bloomington, IN, USA) versus a 22G Franseen needle (Acquire; Boston Scientific, Marlborough, MA, USA) [9]. The mean cumulative length of tissue core biopsies per needle pass was significantly higher with the 22G Franseen needle at 11.4 mm (95% confidence interval [CI] 9.0–13.8) versus 5.4 mm (95% CI 3.8–7.0) for the 20G reverse-bevel needle (*P* < 0.001), and the diagnostic accuracy was also better in the Franseen needle group. Crino et al. performed a randomized, controlled study comparing fork-tip (SharkCore; Medtronic, Dublin, Ireland) or reverse-bevel 22G or 25G needles in 129 patients [10]. The primary outcome was histologic yield. In this study, both needles showed equivalent safety and diagnostic accuracy. However, fork-tip needles provided a higher rate of good-quality histologic samples and required fewer needle passes to reach a diagnosis. Bang et al. performed a randomized trial of 129 patients with pancreatic masses randomized to sampling with reverse-bevel, Menghini-tip (EZ Shot; Olympus, Tokyo, Japan), Franseen, or fork-tip needles [11]. The study also randomized the technique to suction, no suction, and stylet retraction, and the primary endpoint was the core tissue collection rate. The results showed higher cellularity with the fork-tip or Franseen needle than with the other two needles. Based on the results of these studies, the reverse-bevel needle was positioned as a second-generation needle, while the fork-tip and Franseen needles are now considered third-generation needles. Bang et al. also performed a randomized trial in 50 patients with SPLs, using both the 22G Franseen and 22G fork-tip needles for each case and randomizing the order in which they were used [12]. The primary endpoint was a comparison of tissue area, which included 44 cases of pancreatic cancer with no significant difference in total tissue area (6.1 vs. 8.2 mm^2^) or positive diagnosis rate (94% vs. 98%). Ashat et al. performed a randomized trial in 134 patients with 150 lesions randomized 1:1 to the Franseen or fork-tip needle, of which 75 lesions were pancreatic lesions [13]. The primary endpoint was the tissue collection rate, with no significant difference in the rate of adequate tissue sample collection (94.7% vs. 96%, *P* = 1.00) and no significant difference in the rate of positive histological examinations (85.3% vs. 90.7%, *P* = 0.45).

**Table 1:** Randomized studies comparing core needles for EUS-FNB of solid pancreatic lesions

Year	Author	Country	Main Outcome	Needles	Gauge of needles	Number of cases	Result
2020	Karsenti et al. [9]	France	Mean cumulative length of tissue core biopsies	Franseen Reverse-bevel	22G 20G	60	Franseen needle provided more tissue for histologic evaluation and better diagnostic accuracy
2020	Crino et al. [10]	Italy	Histologic yield	Fork-tip Reverse-bevel	22G and 25G	192	Fork-tip needles provided a higher rate of good-quality histologic samples
2018	Bang et al. [12]	US	Area of histologic core tissue	Franseen Fork-tip	22G	50	No significant difference between Franseen and fork-tip needles in terms of area of total tissue
2021	Bang et al. [11]	US	Cellularity of the samples	Reverse-bevel Menghini-tip Franseen Fork-tip	22G	129	Fork-tip or Franseen needles had significantly higher cellularity
2021	Ashat et al. [13]	US	Quality of the tissue samples	Franseen Fork-tip	22G	150	No significant difference between Franseen and fork-tip needles in terms of the yield of adequate histologic samples

*EUS-FNB* endoscopic ultrasound-guided fine-needle biopsy

The present authors also previously examined the usefulness of the fork-tip needle and reported a positive diagnosis rate of 90.6% in 85 cases, and that the diagnostic performance of the 25G needle, which is thinner than the 22G needle, was comparable to that of the 22G needle [14]. In addition, the authors retrospectively reviewed 87 consecutive EUS-FNB specimens using either a 22G Franseen needle (Group A, *N* = 51) or a conventional 22G FNA needle (Group B, *N* = 36) for pancreatic diseases. Although the diagnostic accuracy for malignancy was 96.1% in Group A versus 88.9% in Group B, with no statistically significant difference (*P* = 0.19), the median sample area was significantly larger in Group A (4.07 vs. 1.31 mm^2^, *P* < 0.0001), with no significant difference in adverse events [15].

Autoimmune pancreatitis (AIP) is one of the pancreatic diseases in which pathological diagnosis based on specimens collected via EUS-TA is particularly important [16], and the evolution of needles plays a significant role in the diagnosis of AIP. Many reports have been published on the ability of EUS-TA to enable the histological diagnosis of AIP [17–20], and using EUS-FNB needles in the diagnosis of AIP has become the gold standard in recent years. The authors reported in a prospective multicenter study that EUS-FNB using a 22G Franseen needle demonstrated high diagnostic performance in the histological diagnosis of AIP [17], which made it possible to obtain definitive histological diagnosis in 32 out of 55 (58.2%) patients, which was apparently higher than our historical results (7.9%) using a conventional FNA needle.

Based on these results, the Franseen or fork-tip needle tends to be used as the first choice for EUS-FNB in current practice. In fact, the most recent network meta-analysis of end-cutting EUS-FNB needles for SPLs reported that the Franseen (surface under the cumulative ranking score, 0.89 for accuracy and 0.94 for adequacy) and fork-tip needles (surface under the cumulative ranking score, 0.76 for accuracy and 0.73 for adequacy) ranked as the two highest-performing FNB needles [21].

More recently, several new FNB needles have emerged, such as a needle with three symmetrical heels (Franseen shape) with an inner cut added to the previous design (SonoTip TopGain; Medico’s Hirata, Osaka, Japan) or a needle with a multiblade three-prong tip, which is called a trident-shaped needle (Trident; MICRO-TECH, Ann Arbor, MI, USA). Compared to conventional needles, these new needles may have improved puncture performance, and their usefulness is expected.

## Number of actuations (to-and-fro movements)

The evolution of needles has made it easier to obtain tissue than in the past, and it has been suggested that the number of actuations of the needle can be reduced.

Takahashi et al. reported a multicenter, randomized, controlled trial comparing three and 12 actuations per pass of EUS-FNB [22]. A 22G Franseen needle was used in all patients, and 220 punctures were performed in 110 patients. The diagnostic sensitivity of the three-actuation group, which was the primary endpoint of this study, was noninferior to that of the 12-actuation group (88.6% vs. 89.5%; difference, − 0.9%; 95% CI − 9.81 to 7.86). In addition, the Macroscopic Visual Quality Evaluation Score, which is used to calculate the percentage of blood clots on the slide, was used in this study, and the percentage of patients with a score of 3 or higher was significantly higher in the three-actuation group (71.8% vs. 52.7%, *P* = 0.009). The conclusion of the study was that there was less blood contamination in the three-actuation group, which may facilitate specimen processing and reduce the burden on pathologists.

The present authors also conducted a study at Nagoya University Hospital on the number of actuations, in which the number of actuations was randomly assigned to five and 15 times [23]. This was also a noninferiority study, but in this study, the positive histological examination rates of the 15-actuation group and the five-actuation group were 83.5% (71/85) and 77.7% (66/85), respectively, showing no noninferiority (95% CI − 15.6 to 3.4) of the five-actuation group over the 15-actuation group. Moreover, the 15-actuation group was significantly superior in terms of tissue area and cytological evaluation. Overall, there was no difference in histological diagnostic performance, and 15 actuations may be preferable to five actuations in terms of the possibility of obtaining more core tissue and ease of cytological diagnostic evaluation.

Based on the above, with the advent of the FNB needle, there is a high possibility that diagnosis can be made with even fewer to-and-fro movements, but further research may be needed to determine the optimal number of actuations.

## Methods of sampling at the time of puncture

There has been ongoing research on multiple techniques to increase the diagnostic performance and tissue yield of EUS-TA (Table 2). These include the door-knocking method and fanning technique for actuations after the puncture. Mukai et al. conducted a multicenter, prospective trial in 82 patients who had SPLs and underwent EUS-FNA with the conventional method and door-knocking method with two respective passes in turn [24]. It was found that EUS-FNA with a 22G needle and the door-knocking method did not improve the accuracy of histological diagnosis but enabled acquisition of a larger amount of tissue based on transgastric puncture. Lee et al. performed a prospective comparative study investigating EUS-FNA performed for SPLs, and pairwise specimens were alternately obtained using the two techniques of standard suction or slow pull with fanning [25]. Forty-eight consecutive patients were included in this study, and the slow pull with Fanning technique had a significantly superior diagnostic accuracy than the suction technique (88% vs. 71%, *P* = 0.044). Most recently, Yang et al. proposed a novel torque technique for EUS-FNB [26]. They conducted a randomized trial with 160 patients who underwent EUS-FNB for SPLs using either the torque or fanning technique with the standard technique as a reference. The median quality score of the histological samples was significantly higher in the torque and fanning group than in the standard group (*P* < 0.001). Furthermore, the torque technique provided improved sensitivity of 93.38% and accuracy of 94.30%. Therefore, the new torque technique enables the acquisition of better-quality samples and can potentially increase the diagnostic outcomes in the EUS-FNB of SPLs, with the same recommendations as those for the fanning technique.

**Table 2:** Puncture techniques in EUS-FNA/B

Techniques	Description
Conventional method	Conventional to-and-fro movements of the needle as fast as possible within the lesion after needle puncture
Door-knocking method	A maximally quick needle advancement within the target lesion after needle puncture that makes the big knocking sound between the slider and the stopper
Fanning technique	To-and-fro movements of the needle aimed at the different targets within the mass using the up/down knob of the endoscope during each pass
Torque technique	Twisting the body of the echoendoscope to the right (clockwise) or left (counterclockwise) without using the left/right control knob

*EUS-FNA* endoscopic ultrasound-guided fine-needle aspiration, *EUS-FNB* endoscopic ultrasound-guided fine-needle biopsy

The other methods used to collect tissue at the time of puncture include the suction method, in which negative pressure is applied with a syringe [27]; the slow-pull method, in which the stylet is slowly withdrawn [28]; and the wet-suction method, in which the lumen of the needle is filled with saline and negative pressure is applied. A meta-analysis of randomized controlled trials (RCTs) of slow pull and suction for SPLs reviewed seven RCTs with a total of 475 cases (163 lesions biopsied using suction, 164 lesions using slow pull, and 148 lesions using both methods) [29]. In this meta-analysis, there was no difference in the specimen collection rate between the two methods (OR = 0.98), but the suction method was associated with more blood clots (27.6% vs. 19.7%). However, the slow-pull method was not superior to the suction method in terms of the positive diagnosis rate. This analysis suggests that there is no significant difference between the slow-pull and suction methods but that the slow-pull method seems to be better because it produces fewer blood clots than the suction method. In 2023, a multicenter, randomized, crossover trial comparing wet suction and slow pull [30]. In this study, a 22G fork-tip or Franseen needle was used, and a total of four punctures were made with the same needle. The patients were assigned to the wet-suction first or slow-pull first group, and the procedure was performed alternately. In this study, the tissue integrity score was significantly higher in the wet-suction group (*P* = 0.02), but the percentage of blood clots was also significantly higher (*P* < 0.001). On the other hand, the percentage of positive examinations and the percentage of tissue sampling (adequate tumour fraction) were comparable. In other words, the wet-suction method showed a higher core tissue collection rate, but there was no difference in diagnostic performance or tissue collection rate, while the wet-suction method showed a higher blood clot content rate. Lin et al. evaluated the usefulness of heparinized wet suction during EUS-FNB of SPLs [31]. A prospective, randomized, crossover study was conducted in a total of 50 patients with 200 specimens to compare heparinized wet-suction and dry-suction methods. The heparinized wet-suction approach yielded specimens with longer aggregated white tissue length (11.07 mm vs 7.96 mm, *P* = 0.001) and less blood contamination (*P* = 0.008). In addition, the amount of extracted DNA correlated positively to the white tissue, and they mention that heparinized wet suction may allow for next-generation sequencing in CGP tests. Further study is needed to determine the optimal sampling method.

## Combination with contrast-enhanced harmonic EUS

Recently, contrast-enhanced harmonic EUS (CH-EUS) has enabled detailed hemodynamic evaluation of pancreatic lesions [32–34]. More recently, it has been reported that CH-EUS may be combined with EUS-TA to improve diagnostic performance [35–38]. Hou et al. reported that the percentage of adequate biopsy specimens obtained using FNA in a CH-EUS group (96.6%) was greater than that obtained in an EUS-FNA group (86.7%) [36]. Kamata et al. reported that EUS-FNA has lower sensitivity for pancreatic adenocarcinoma with avascular areas on CH-EUS [38], and Itonaga et al. reported that the adequate sampling rate and sensitivity of EUS-FNA for SPLs were significantly higher in combination with CH-EUS (84.9% vs 68.8%, *P* = 0.003 and 76.5% vs 58.8%, *P* = 0.011, respectively) [37]. EUS-TA may yield false-negative results, especially for small pancreatic lesions [35]. In such cases, CH-EUS may contribute to improving diagnostic performance, and further studies are expected.

## Specimen evaluation methods

In 2011, the usefulness of rapid on-site cytopathological evaluation (ROSE) was reported as a specimen evaluation method for EUS-FNA. Iglesias-Garcia et al. reported that ROSE significantly improved the sensitivity (96.2 vs. 78.2%, *P* = 0.002) and accuracy (96.8 vs. 86.2%, *P* = 0.013) of cancer diagnoses and was associated with a significantly lower number of inadequate samples (1.0 vs. 12.6%, *P* = 0.002) and a lower number of needle passes (3.5 ± 1.0 vs. 2.0 ± 0.7, *P* < 0.001) [39]. However, the number of facilities where ROSE can be performed is limited, and it does not necessarily lead to a reduction in testing time. In addition, due to the advent of new core needles, much more tissue can be obtained with a smaller number of needle passes, and some reports suggest that using ROSE to reduce the number of needle passes may be needless in the era of EUS-FNB [40–42]. On the other hand, macroscopic on-site evaluation (MOSE), which visually determines the presence or absence of white core specimens, has been reported to be useful as an alternative to ROSE [43, 44]. In 2020, Kaneko et al. reported the usefulness of MOSE in 75 cases of EUS-FNB for pancreatic masses using a 22G Franseen needle [45]. In this study, the white tissue of the obtained specimens was collected and measured for length. The median length of the visible cores was 15 mm, and these cores were significantly longer in the correct diagnosis group than in the incorrect diagnosis group (7 mm). The accuracy correlated positively with the visible core length, and the receiver operating characteristic curve analysis of the visible core length for accuracy demonstrated an optimal cut-off value of 10 mm.

In 2023, a 10-centre randomized trial from Italy included 370 patients with 234 pancreatic lesions randomly assigned to the EUS-FNB with MOSE group or the EUS-FNB group; three needle passes were performed, and a core sample of 10 mm or longer was considered a MOSE-positive result [46]. The results showed no difference in the positive examination rate, specimen collection rate, or adverse events, but the median number of punctures was significantly lower in the MOSE group (1 vs. 3, *P* < 0.001).

The present authors have previously reported the usefulness of MOSE using a stereomicroscope, namely, S-MOSE [47]. In a study of 60 cases in which both ROSE and S-MOSE were performed, the concordance rate between S-MOSE and tissue diagnosis was 90% (54/60), and the positive predictive value of S-MOSE was 90.7%, which was comparable to the 89.5% positive predictive value of ROSE. We believe that the use of a stereomicroscope in MOSE has three advantages. First, it facilitates the differentiation between blood clots and core tissue by simply magnifying the image compared to the usual macroscopic observation. Second, the stereomicroscope can be installed next to the EUS room, allowing observation immediately after specimen collection, which is expected to shorten the examination time. Finally, all specimens can be evaluated under the same conditions by adjusting the magnification using a scale under stereomicroscopic observation. This increases objectivity, facilitates measurement of tissue length, and will lead to image-based analysis in the future.

These results indicate that MOSE is a simple and useful rapid specimen evaluation method.

## Usefulness of AI diagnostics

In recent years, artificial intelligence (AI)-based systems have evolved and been introduced into medical practice, and in the field of EUS-TA, AI is expected to be utilized in the real-time assessment of sample adequacy and detection of cancer cells in the obtained specimens. Studies on the application of AI models in pathology have focused on gynaecological samples, but there have not yet been many studies on pancreatic EUS-TA, although several reports have been published in the past few years (Table 3).

**Table 3:** Reports on AI diagnosis in EUS-FNA/B of pancreas

	Year	Author	Country	Outcome	Model	Number of cases	Diagnostic performance
EUS-FNA (cytology)	2022	Lin et al. [48]	China	Detection of malignant cells on EUS-FNA specimens for ROSE	DL	51	Accuracy 83.4%
	2022	Zhang et al. [49]	China	Identify cancer cell clusters on EUS-FNA specimens for ROSE	DL (DCNN)	194	AUROC 0.929
EUS-FNB (histology)	2021	Naito et al. [51]	Japan	Differentiation between PC and benign on EUS-FNB specimens	DL (CNN)	532	Accuracy 94.2%
	2022	Ishikawa et al. [50]	Japan	Appropriateness of EUS-FNB specimens	DL (Contrastive learning)	173	Accuracy 84.4%

*AI* artificial intelligence, *EUS-FNA* endoscopic ultrasound-guided fine-needle aspiration, *EUS-FNB* endoscopic ultrasound-guided fine-needle biopsy, *ROSE* rapid on-site evaluation, *PC* pancreatic cancer, *DL* deep learning, *DCNN* deep convolutional neural network, *CNN* convolutional neural network, *AUROC* area under the receiver operating characteristic curve

In EUS-FNA, AI is expected to be utilized in ROSE. Lin et al. developed a new AI-based model (ROSE-AI model) and reported the validation results of its substitution for ROSE during EUS-FNA [48]. The model trained and validated the AI algorithm using digital images acquired from slides of EUS-FNA specimens collected from 51 cases in which pancreatic diseases accounted for 94.1%, with a particular emphasis on the specificity of malignant cells. As a result, the ROSE-AI model achieved a positive diagnosis rate of 83.4% on the internal validation data and 88.7% on the external test data. The study shows that the use of AI is feasible for replacing conventional ROSE during EUS-FNA and suggests that the ROSE-AI model can be used to solve the problem of a shortage of cytologists and make ROSE available to more institutions.

Zhang et al. developed a deep convolutional neural network (DCNN) system using 5345 cytology slide images from 194 patients to identify cancer cell clusters [49]. The DCNN system was superior to trained endoscopists and comparable to cytologists. The DCNN system in this study demonstrated its practicality for identifying the suitability of EUS-FNA specimens and pancreatic cancer cell clusters.

The present authors have been investigating the capability of automated diagnosis via AI using deep learning (DL) for the histological diagnosis of EUS-FNB specimens in pancreatic diseases [50]. This study was conducted as a collaborative project between the Department of Electrical and Electronic Engineering of Meijo University and Nagoya University Hospital. At the 52nd Annual Meeting of the Japanese Pancreas Society in 2021, we reported on the feasibility of histological diagnosis of EUS-FNB specimens using DL based on stereomicroscopic images. The diagnostic performance of AI using data from 98 specimens was 71.8%, which was not comparable to the 81.6% diagnostic performance of MOSE performed by EUS experts. When the number of specimens was increased to 173, the diagnostic performance increased to 74.5%, but this result was not satisfactory. On the other hand, AI analysis using haematoxylin eosin (HE)-stained slides increased the sensitivity, specificity, and positive predictive value, with the positive predictive value being higher than that of MOSE. Based on this finding, we decided to conduct a further study to improve diagnostic performance by using contrastive learning. Contrastive learning is a type of unsupervised learning in which a large amount of data are learned by comparing data without labelling. We constructed a new network model incorporating contrast learning and evaluated EUS-FNB specimens with this model. The model learned the relationship between HE-stained images and marked tissue images and identified which pixel corresponded to the tissue. As a result, the overlap rate (IoU) between HE-stained and marked images was 89.6%. Furthermore, we attempted to predict the area of the tissue and to see if the AI could improve its diagnostic ability by learning the features of the stereomicroscopic image and the HE-stained image to be closer to each other. In this model, multiple networks were trained simultaneously, and only some of the networks were used for inference. We also performed 8-part cross-validation for each of the 145 diagnosable and 28 nondiagnosable samples. As a result, when compared to MOSE, AI with contrast learning showed equal or better predictive ability with a sensitivity of 90.3%, specificity of 53.5%, positive predictive accuracy of 84.4%, positive predictive accuracy of 90.9%, and negative predictive accuracy of 51.7%. To further improve the accuracy, especially the specificity, we plan to explore and improve more effective learning methods for problems with a small number of images that are not available for testing.

The introduction of digitized whole-slide image (WSI) technology has enabled the application of digital pathology in clinical and educational settings. This digitization has facilitated the implementation of AI-based algorithms in the field of pathology. In 2021, Naito et al. developed a DL model for diagnosing pancreatic cancer using EUS-FNB specimens [51]. Preoperative pathological diagnosis using EUS-FNB has become the mainstream in pancreatic cancer treatment. However, accurate histopathological evaluation of EUS-FNB specimens is sometimes difficult due to the small sample volume and contamination of blood and inflammatory/gastrointestinal cells. In the study by Naito et al., 532 WSIs were annotated with training sets by pancreatic pathologists, DL models were trained, and EUS-FNB specimens of pancreatic cancer were evaluated with WSI. As a result, the model obtained a high evaluation performance with an AUROC of 0.984, positive diagnosis rate of 94.2%, sensitivity of 93%, and specificity of 97%. This model is capable of accurately detecting even difficult cases in which the number of cancer cells in the specimen is small and is reported to be an aid to pathologists in difficult-to-diagnose cases.

The ideal AI model for EUS-TA would be something that can provide highly accurate diagnoses of benign or malignant conditions even for small amounts of specimens or specimens that contain blood clots and that can provide such information in real time during the examination. The development of AI models in EUS-TA will be a major step forwards in the treatment of pancreatic cancer and other pancreatic diseases. If this model is refined and made available in clinical practice, it may further improve the diagnostic accuracy of pancreatic EUS-TA.

## EUS-TA in comprehensive genomic profiling (CGP)

Optimal sampling beyond diagnostic accuracy is important to enable CGP for cancer treatment. To achieve this goal, it is necessary to increase the quantity and quality of the core tissue obtained via EUS-FNB. However, in previous studies performed at Nagoya University Hospital using a 22G Franseen needle [23, 47], only approximately 2 mm^2^ was obtained from correctly diagnosed core tissue, which is much smaller than the area required by the Foundation One CDx (Foundation Medicine, Cambridge, MA, USA) (25 mm^2^), one of the commercially available CGP tests [23].

Increasing the needle gauge and number of passes may be necessary to achieve this goal. It has been reported that 19G Franseen needles can increase the area by approximately three times compared to 22G needles [52]. Hisada et al. performed EUS-TA using a 19G FNB needle in 33 patients with unresectable pancreatic cancer and investigated the results of CGP testing on the obtained specimens [53]. The purpose of the study was to evaluate the percentage of specimens that met the eligibility criteria for OncoGuide TM NCC Oncopanel System (NOP; Sysmex Corporation, Hyogo, Japan) analysis. The procedure success rate was 100%, and a class V cell diagnosis was confirmed in all patients. The percentage of patients fulfilling the eligibility criteria for NOP analysis was 63.6%, indicating that EUS-TA using a 19G FNB needle is a valid test method that fulfils the eligibility criteria for NOP analysis.

On the other hand, it has been reported that measurement of core tissue length can predict whether a specimen is suitable for panel testing [54]. A 22G Franseen needle was used in this study, and the Foundation One CDx was used as the CGP test. It was assumed that if there were more than six fields of view in a 10× field of view, there was more than 25 mm^2^ of tissue in the calculation, and the receiver operating characteristic (ROC) analysis was based on the length of the gross core tissue. The cut-off value for the CGP was 30 mm, and the area under the ROC curve was 0.74 (95% CI 0.65–0.83). This study indicates that even a 22G needle can collect specimens for CGP if a sufficient length of core tissue (30 mm or more as a rough guide) is collected. Further studies are needed to determine the optimal needle shape and gauge, specimen volume, and specimen processing methods for CGP.

## Conclusions

In this article, we reviewed the cutting edge of EUS-TA, including EUS-FNA and EUS-FNB, for SPLs. Needles evolved and changed their shape to collect more specimens for EUS-TA. In the era of EUS-FNB for histological diagnosis, MOSE is a simple and useful rapid specimen evaluation method, and the measurement of core tissue length in MOSE can be a useful indicator for evaluating specimen quality, especially for determining whether CGP is available. Several new needles are emerging, which are expected to further simplify the procedure and improve diagnostic performance.

## Data Availability

The data of this study are available from the corresponding author upon reasonable request.
